# Experiential Learning and Mentorship in Global Health Leadership Programs: Capturing Lessons from Across the Globe

**DOI:** 10.5334/aogh.3194

**Published:** 2021-07-12

**Authors:** Daniela C. Rodríguez, Nasreen S. Jessani, Joseph Zunt, Sara Ardila-Gómez, Patience A. Muwanguzi, Sylvester Ndeso Atanga, Bruno Sunguya, Carey Farquhar, Esther Nasuuna

**Affiliations:** 1 Johns Hopkins Bloomberg School of Public Health, USA; 2 STAR Project, USA; 3 Centre for Evidence Based Health Care, Stellenbosch University, South Africa; 4 Emerging Voices for Global Health; 5 University of Washington, Seattle, USA; 6 Fogarty Global Health Fellows Program, USA; 7 School of Health Sciences, Makerere University College of Health Sciences, Kampala, Uganda; 8 Afya Bora Consortium; 9 Saint Monica American International University; 10 School of Health & Human Services, Buea, Cameroon; 11 Muhimbili University of Health and Allied Sciences, Tanzania; 12 Departments of Global Health, Medicine and Epidemiology, University of Washington; 13 Infectious Diseases Institute, Makerere University College of Health Sciences, Kampala, Uganda

## Abstract

**Objectives:**

The changing global landscape of disease and public health crises, such as the current COVID-19 pandemic, call for a new generation of global health leaders. As global health leadership programs evolve, many have incorporated experiential learning and mentoring (ELM) components into their structure. However, there has been incomplete consideration on how ELM activities are deployed, what challenges they face and how programs adapt to meet those challenges. This paper builds on the co-authors’ experiences as trainees, trainers, organizers and evaluators of six global health leadership programs to reflect on lessons learned regarding ELM. We also consider ethics, technology, gender, age and framing that influence how ELM activities are developed and implemented.

**Findings:**

Despite the diverse origins and funding of these programs, all six are focused on training participants from low- and middle-income countries drawing on a diversity of professions. Each program uses mixed didactic approaches, practice-based placements, competency and skills-driven curricula, and mentorship via various modalities. Main metrics for success include development of trainee networks, acquisition of skills and formation of relationships; programs that included research training had specific research metrics as well. Common challenges the programs face include ensuring clarity of expectations of all participants and mentors; maintaining connection among trainees; meeting the needs of trainee cohorts with different skill sets and starting points; and ensuring trainee cohorts capture age, gender and other forms of diversity.

**Conclusions:**

ELM activities for global health leadership are proving even more critical now as the importance of effective individual leaders in responding to crises becomes evident. Future efforts for ELM in global health leadership should emphasize local adaptation and sustainability. Practice-based learning and established mentoring relationships provide the building blocks for competent leaders to navigate complex dynamics with the flexibility and conscientiousness needed to improve the health of global populations.

**Key Takeaways:**

- Experiential learning and mentorship activities within global health leadership programs provide the hands-on practice and support that the next generation of global health leaders need to address the health challenges of our times.
- Six global health leadership programs with experiential learning and mentorship components are showcased to highlight differences and similarities in their approaches and capture a broad picture of achievements that can help inform future programs.
- Emphasis on inter-professional training, mixed-learning approaches and mentorship modalities were common across programs. Both individual capacity building and development of trainees’ professional networks were seen as critical, reflecting the value of inter-personal connections for long-term leadership success.
- During program design, future programs should recognize the “frame” within which the program will be incorporated and intentionally address diversity—in all its forms—during recruitment as well as consider North-South ethics, leadership roles, hierarchies and transition plans.

## Introduction

As the global health landscape changes, new conceptualizations about the skills global health leaders require to pursue health goals are being debated and the image of desk-bound bureaucrats as primary decision-makers is being contested [1]. The next generation of global health leaders is emerging from various disciplines and they need to experience what is happening in the field if they are to act and react appropriately, especially during times of crisis. In line with this, efforts to strengthen leadership in global health through experiential learning and mentorship (ELM) are growing through both structured programs and informal activities that contribute to building a deeper field of professionals. There is considerable guidance on how to do ELM but there is less information on review and reflection of existing programs [2]. We have had the privilege of participating, as trainers, organizers, participants, and evaluators, in global health leadership training programs that represent a variety of modalities and participant profiles. In this paper, we draw on our practice-based experiences to reflect on what works in ELM for developing global health leaders. We close by presenting core principles to guide ELM activities that address partnership and collaboration between the global South and North. To ground the discussion, we first define ELM and highlight the importance of these approaches in providing practical learning.

**Experiential learning** is a process of acquiring skills, knowledge and experience through firsthand engagement within a competency or vocation. It places the locus of learning close to the site of application [3]. There are four stages in experiential learning: experience, reflect, conceptualize and experiment [4]. Kolb’s Experiential Learning Theory purports that “knowledge results from a combination of a) grasping the experience through concrete experience and abstract conceptualization; and b) transforming the experience through reflective observation and active experimentation” [5]. The kind of experience that a global health leader acquires defines the leadership skills that will be developed. Leaders who are exposed to a variety of experiences are usually more effective [6].

Research from business and psychology disciplines indicates that linear sequential approaches to leadership development tend to fail, in contrast to those that employ a more complex, responsive, adaptive and practical process [7]. Experience-based leadership development engages future leaders personally and captures the broader systems context while using trainers “who act less as experts and more as Sherpas” in order to “guide participants toward their personal and organizational summits” [8].

In order to ensure that the next generation of global health leaders has opportunities for experiential learning, a range of service providers has appeared in the learning landscape, including institutes of higher education, government and non-government organizations, digital startups and management consulting firms. Short-term experiential opportunities include simulations of real-world scenarios, such as mock World Health Assembly within formal graduate studies; internships at global health implementing and decision-making agencies; site attachments at research and implementing organizations and Ministries of Health; apprenticeships at key health policy departments; project-based assignments, field research, sabbaticals, and/or community service learning projects. Increased attention from national and international donors has also led to an emergence and evolution of stand-alone programs dedicated to experiential training in global health leadership.

We define **mentorship for global health leadership** as the support and guidance of skills, social development and reflection that contributes to the transformative growth of an individual’s global health practice, research and professionalism. Successful mentorship can strengthen not only an individual mentee but their networks and affiliated institutions, and build long-term collaborations [2]. The value of “network intelligence” as key to leadership advancement has been widely acknowledged [9]. We have not limited our interpretation of mentoring to any specific modality (e.g., in matched pairs, between peers, at a distance, etc.) [10] because we believe the principles described here apply regardless of approach and reflect how an individual’s mentorship experience can vary over time.

Similar to other experiential training programs, those focusing on global health leadership depend on mentors who have the relevant experience and skills to be good mentors, and the time to dedicate to mentoring. Challenges to mentorship for global health include [11] (i) operating within hierarchical environments that affect mentoring relationships and interactions between local and foreign professionals that may create tensions [12], (ii) limited measures for assessing success or ensuring accountability [13], (iii) difficulties identifying, incentivizing and retaining mentors with sufficient time and experience to dedicate to mentees, (iv) lack of (or inadequate) training and guidance on how to be a responsive mentor, (v) assumptions that mentoring needs to be a long formal relationship, and (vi) assumptions that seniority is required—thus precluding mentoring from peers. In the long run, maintaining successful mentoring relationships beyond a formalized program poses further challenges in continuity, sustainability and growth.

By focusing on cross-country experiences and learning, global health leadership programs create opportunities for trainees with different backgrounds and experiences to interact and learn from their approach to similar problems. Although the range of skills and competencies required for effective global health leadership [5] is wide, ELM contributes to building an individual’s strengths and recognizing areas for improvement. However, ELM suffers from gaps in global health leadership programs that have persistently gone unaddressed. First, these programs can be too narrow, often due to restrictions from funding sources (e.g., focused on one type of global health professional or one health topic), and these limitations undermine the interdisciplinary needs of global health. The sustainability of leadership programs is also threatened when the funding base is linked to timelines of donor or project-based financing, although there are exceptions (see ***Box 1***).

**Box 1 Kuskaya Program, Peru**

The Kuskaya Program in Peru [14] was funded by the “Innovations in Global Health” initiative directed by the NIH Fogarty International Center and awarded to the Universidad Peruana Cayetano Heredia, with the University of Washington as a collaborating partner. The program focused on training Peruvian investigators to be ethical and effective mentors in a systematic and standardized manner. Ethical conduct of research requires a) mentors with high ethical standards and b) training programs in the responsible conduct of research (RCR). Institutionalization of mentorship and RCR training facilitates adaptation to local languages, customs and populations, with greater dissemination into successive generations of emerging scientists.

The Universidad Peruana Cayetano Heredia (UPCH), with support from a variety of research training grants provided by the US NIH Fogarty International Center, later developed an online course on RCR and mentorship training “Conducta Responsable en Investigación” (*http://www.cri.andeanquipu.org*). This course contains seven modules in RCR and mentoring and culminates in a certificate provided by either UPCH or the Peruvian government’s Consejo Nacional de Ciencia, Tecnología e Innovación Tecnológica (CONCYTEC). The Kuskaya Program is therefore an example of a successfully institutionalized and locally sustained program.

There is a need to update the current learning model that sequences “education prior to work experience” to a model where education and work experience are complementary parts of a training whole. One such program is the ROPES program that was adapted from the OUTWARD Bound training program [15]. It has been used by over 30 companies in the United States in their leadership programs for managers. It was specifically used at the East Carolina University by the senior nursing leadership course to teach leadership skills to nurses through a combination of psychological and physical problem-solving activities inside and outside the classroom. These are designed in a way to enable participants to gain self-confidence, build teamwork and push their personal boundaries [15]. Global health leadership training should also be shifting away from structured curricula to experiential and problem-solving learning approaches to ensure trainees are prepared for the multiple permutations of health systems and programs they will encounter as leaders. Lastly, leadership training programs for global health need to grapple with the ethics and power dynamics between countries, especially North-South partnerships, how they contribute to perpetuating these (e.g., opportunities for brain drain), and what role programs and trainees can play in dismantling these historical imbalances.

The short-term nature of many such programs has limited the reflection, documentation and dissemination of successful models, results, outcomes and learnings, as well as common challenges encountered. We also see ELM programs repeatedly re-inventing the wheel on what content to include and how to structure training efforts, leading to redundancy and duplication. Our paper begins to address this specific challenge by capturing snapshots of ELM activities within six global health leadership programs, comparing their approaches and reflecting on lessons from the field.

## Experiential Learning and Mentoring within Global Health Leadership Programs

Here we present the experiences of six global health leadership programs that have incorporated experiential learning, mentorship or both in innovative ways. While we acknowledge the existence of other ELM programs, the programs we describe reflect our in-depth knowledge and experiences with these programs, along with other co-authors of this series, as participants, organizers, evaluators or a combination of the aforementioned roles.

- Afya Bora Global Health Fellowship Program: a one-year fellowship for health professionals from five sub-Saharan African countries (Tanzania, Kenya, Uganda, Botswana and Cameroon), China and the U.S. that fosters skills in leadership through South-South and North-South collaborations. Afya Bora targets professionals with medical, nursing and public health backgrounds with the goal to prepare trainees to address global health challenges, especially HIV/AIDS, while preparing them to be future leaders in their respective organizations and beyond. It was started in 2009 and funded by Health Resources and Services Administration (HRSA), PEPFAR and the National Institutes of Health (NIH) Office of AIDS Research and Fogarty International Center [16].
- Chatham House Leadership Program: fellowship targeting Africans with leadership potential working in public health in Africa to develop skills in leadership, policy analysis and formulation. Started in 2016 with funding from the Rockefeller Foundation; currently the main funder is the International Federation of Pharmaceutical Manufacturers and Associations.
- Collaboration for Health Policy and Systems Analysis in Africa (CHEPSAA): Funded by the European Union, CHEPSAA was a consortium of African and European universities with the overall purpose of increasing sustainable African capacity to produce and use high quality Health Policy and Systems Research and Analysis (HPSR+A). The Emerging Leaders Programme (ELP) ran from approximately 2013–2015 and comprised two rounds of capacity development workshops, mentoring during the workshops and in the periods between workshops, and participation in the Third Global Symposium on Health Systems Research, which took place in Cape Town in 2014. The ELP was supported with CHEPSAA’s project funds, as well as additional funds from the Rockefeller Foundation [17].
- Emerging Voices for Global Health (EV4GH): Conceived in 2010 by the Institute of Tropical Medicine in Antwerp, EV4GH targets young emerging health policy & systems researchers, decision-makers, and other health system professionals from the global South (65 countries to date), with an interest in becoming influential global health voices and/or local change makers. It is an innovative multi-partner blended training program that includes (biennial) face-to-face training and virtual training components. Since 2014 the program is alumni-led, with a governance board with representatives from different world regions and a secretariat based at the Institute of Public Health, Bengaluru, India (IPH) [18].
- Fogarty Global Health Fellows, Northern Pacific Global Health Fellows consortium (Fogarty-NP) [19]: a one-year clinical research training program targeting doctoral and post-doctoral health professionals from nine low- and middle-income countries (LMICs) (Cameroon, Ghana, India, Kenya, Liberia, Nepal, Peru, Thailand and Uganda). Trainees receive mentorship and training and perform a research global health project of their choosing. Funded by the National Institutes of Health’s Fogarty International Center, Fogarty-NP started in 2012.
- Sustaining Technical and Analytic Resources (STAR): a project focused on capacity building of global health professionals through multiple mechanisms. In this paper we focus on STAR fellows, who are mid-to-senior career professionals providing technical support in the US and globally. Started in 2018 and funded by the US Agency for International Development (USAID) [10].

***Table 1*** describes each program, their goals, target audience and considerations arising from their implementation.

**Table 1:** Components, Measurement, Challenges and Lessons Learned of ELM Activities of Global Health Leadership Programs.


PROGRAM NAME	TARGET AND NUMBER OF PARTICIPANTS	INNOVATIONS IN EXPERIENTIAL LEARNING	INNOVATIONS IN MENTORING	PROGRAM OUTCOMES AROUND ELM	METRICS FOR ASSESSING PROGRAM OUTCOMES	CHALLENGES IN IMPLEMENTATION	LESSONS LEARNED FROM IMPLEMENTATION

**Afya Bora Global Health fellowship** *http://www.afyaboraconsortium.org/index.html*	Doctors, nurses, public health specialists 189 fellows since 2011 from Uganda, Kenya, Tanzania, Botswana, and Cameroon, China, USA	Each fellow undergoes two four-and-a-half-month placements at an attachment site. Attachment Sites are organizations that serve as the basis for experiential learning internships for Fellows in the African partner countries. These organizations include both governmental (Ministries of Health) and non-governmental organizations (NGOs), and other international health organizations. The placements allow fellows to integrate into local organizations as well as to network with local and regional actors in the field of global health. While at the attachment site, each fellow is supposed to create a research project that is addressing the observed challenges under the mentorship of the site mentor and primary mentor.	Each fellow has a primary mentor and is assigned a site mentor at the attachment site. There is also an overall country mentor for each cohort. All fellows receive quarterly in-country group sessions or more as required. Each fellow designs a standard meeting schedule with their primary mentor but may request more meetings as needed. While at the attachment site, each fellow has regular meetings with their site mentor depending on the site mentor’s level of involvement with their work.	Individual outcomes: 1. Career advancement 2. Improved performance 3. Increased research skills 4. Professional network broadened 5. Improved communication skills Workplace outcomes 1. Increased leadership capacity 2. Improved efficiency 3. Increased research capacity 4. Sustainable mentorship	Evaluations are conducted regularly (e.g., after each module and apprenticeship), and include self-assessments, feedback through journaling, and competency-based performance checklist. Mentors also provide feedback on performance. At each joint meeting, fellows present their progress, including their projects, and receive feedback from mentors across the countries.	Mentors workshop is held every year after recruiting. In some cases, trained mentors are unable to mentor (e.g., MOH turnover) or mentees require a new mentor, which results in constant need for retraining. Striking a balance between fellows’ regular jobs and the needs of the fellowship, especially when facing funding cuts at their workplace. Location of attachment sites: some recruited fellows are from rural areas or districts, and for them to attend place of attachment and training they have to re-locate to be able to achieve the expectations of the fellowship. Instability of internet connectivity in various countries for distance learning modules. Tracking and follow-up of fellows beyond the fellowship period. Selection of topics and methods may exceed the one year to Implement them, causing partial fulfillment of the targets.	Fellows may be forced to take more time to take the modules or get alternatives on how to download the modules, including downloading content to flash drives for ease of access

**Chatham House Leadership Program**	This fellowship is open to Africans working in Africa who have a background in public health sector management and who possess leadership potential. 14 total (6 per cohort, on average)	A hallmark of the fellowship is its mix of intensive orientation, interaction with leaders in public health and high-level remote mentoring.	Mentoring is provided by leading experts at the Chatham House Centre on Global Health Security in London and at the Leadership Academy of the African Union in Addis Ababa, Ethiopia. The major requirement is that fellows complete a relevant independent project, under virtual mentoring, while maintaining their jobs in their home countries.	1. Strengthen their capacity to develop, implement and evaluate public health initiatives 2. Hone their leadership skills 3. Improve their ability to assess public health in their own country 4. Build networks across relevant sectors.	Fellows are evaluated through their leadership research projects, and leadership development progress through the remote mentorship, measure	Limit in size of cohorts due to lack of physical space. Value for money and cost effectiveness of program.	

**Collaboration for Health Policy and Systems Analysis in Africa (CHEPSAA)** *https://www.hpsa-africa.org/index.php/emerging-leaders-news*	The Emerging Leaders Programme (ELP) was one component of the broader CHEPSAA program. Participation was open to academic staff and public sector officials only from the CHEPSAA African partner countries (South Africa, Tanzania, Kenya, Ghana and Nigeria) who are engaged in the field of HPSR plus academia and who had the potential to develop as leaders in the field over time. 26 emerging leaders (ELs) from across all of CHEPSAA countries including representatives of both the academic partners in CHEPSAA and non-academic partners.	The CHEPSAA ELP was unique because it permitted aspiring leaders to design their own program of capacity building based on their own perceived competency needs. Furthermore, they were provided the funding and professional support from an international consortium to pursue these goals. The ELP consisted of three face-to-face capacity building workshops addressing leadership skills, curriculum development and a special session at the biennial Health Systems Research Symposium.	Leadership training was embedded in the fact that the program coordinator was an EL. ELs were exposed to senior HPSR researchers as well as life coaches who contributed to the hands-on exercises in the capacity building workshops.	1. Training in curriculum development 2. Training in facilitation skills 3. Enhanced personal skills, writing skills, project management skills, networking skills, understanding and knowledge of HPSR&A, understanding of the health system, teaching skills, and research skills. 4. Enhanced networks and collaborations 5. Enhanced understanding about unique approaches to leadership, management and self-realization	1. Leadership: ELs spearheaded a conference session entitled Emerging Leaders in Health Policy and Systems Research: Assuming Leadership in HPSR&A – Personal reflections and lessons. 2. Facilitation and management skills: Prior to the conference, ELs convened face-to-face to finalize the organized panel, decide on ways forward for the ELP, host an expert facilitated session on Mentorship skills, etc. 3. International presentations: ELs presented their experiences from the ELP at various conferences in South Africa and elsewhere. 4. Networks and collaborations: Cross-country EL relationships included analytical support from a Kenyan EL to a Nigerian EL, and planned joint publications between a Ghanaian and S. African ELs.	ELs joining the program with different interests and knowledge of HPSR. Ensuring all ELs felt equal co-ownership of the program was challenging. Varying levels of engagement across the ELs	Varied practices across partners to recruit ELs had led to a highly motivated group of individuals, but also quite a diverse group which sometimes proved difficult when trying to meet different individuals’ expectations of the program. More pressingly, participants voiced considerable concern about the sustainability of the program once CHEPSAA ended, feeling that the program needed a longer timeframe to truly come to fruition. Programs such as these could be of most value for ELs at similar stages in their careers who have similar goals and needs from such a program and can be co-creators in the evolution of the objectives.

**Emerging Voices for Global Health** *http://www.ev4gh.net/*	Health policy and systems researchers, mainly from the Global South. Also includes other health systems actors such as activists, service managers, journalists and health practitioners. Seven cohorts between 2010 and 2020 totaling 290 participants from 65 countries.	The format of the program has evolved over the years. The 2018 program was comprised of a 2-month distance learning program complemented by an intensive 6–10 day face-to-face program that culminates in a preconference prior to the biennial Health Systems Research (HSR) Symposium. The face-to-face program offers practical skill building, peer feedback, coaching, mentoring and production of high quality products for various audiences. Learning includes role plays, simulations, fishbowls, case studies, mock interviews and musical productions. It also includes visit to local health system’s relevant organizations. For each cohort, the program counts with the support of a local partner institution.	For each cohort, the majority of the facilitators are EV4GH alumni who volunteer to plan and execute the program. These facilitators serve as mentors during the venture but often after as well. In addition, the network is supported by junior and senior health policy and systems research (HPSR) researchers from around the world who serve as additional mentors. Peers also serve a critical role in feedback, support and coaching thereby also serving as mentors. Coaching and mentoring occur during both phases of the venture.	1. Enhanced networks and collaborations. 2. Greater opportunities for knowledge sharing. 3. Improved oral, written, networking, leadership, mentoring, collaboration and communication skills. 4. Leadership skills and confidence. 5. Advanced Knowledge Translation skills. 6. Comfort in thinking globally and critically. 7. Commitment to EV4GH’s sustainability.	1. Enhanced networks translate into collaborative writing, joint proposals for funding, invitations to present, etc. 2. Knowledge sharing through engagement in the E44GH program, contributions to the International Health Policies blog, social media posts tagging @EV4GH, sharing through the EV4GH listserv. 3. Empowerment and confidence translates into applications (and successful acceptance) into leadership roles (e.g., selected as plenary speaker for the HSR symposia, journal editorial board, leadership in HPSR society, etc.) 4. Advanced KT skills through utilization of innovative and tailored approaches relevant to different audiences. Winning prizes for best poster at HSR. High-quality, impactful presentations and speeches at HSR (and beyond). 5. Volunteer commitment and capacity: The venture depends on alumni volunteers. The number who provide time and knowledge to driving the cohort is an indicator for ownership, buy-in and value of the program.	Functioning of the network between cohorts to ensure regular engagement and exchange of information. Face-to-face as a core component for the development of enduring networks. Its length is limited by funding constrains. How to include under-represented regions, who do not fall within funders’ categories (e.g., Eastern Europe, some Latin American countries). Visas for participants have become a problem during the last cohorts: some participants miss in-person component due to visa restrictions.	To have diversity in the levels of the candidates has been important, in terms of gender, regions, backgrounds and levels of leadership skills. The program intentionally seeks out those who can benefit from the training and the network, those who have not yet “emerged.” Partner institutions have been fundamental for the delivery of the in-person program by providing the opportunity to think about the local context where global events take place, and to engage with different actors of a given health system.

Fogarty Global Health Fellows* https://www.fogartyfellows.org, *The information in this table pertains to one consortium – the Northern Pacific Global Health Fellows consortium.	Post-doctorate trainees and doctoral students in the health sciences and allied fields. This program funds 6 US-based consortia, each including 4 US academic institutions, with collaborations in more than 35 countries. 178 trainees over past nine years from Cameroon, Ghana, Peru, Kenya, Uganda, Thailand, India, Nepal and Liberia.	Weekly on-line “Core Competencies for Global Health” curriculum provides an opportunity for all trainees to establish competency in eight thematic domains relevant to global health research as they implement, conduct and analyze their research projects. Curriculum learning activities are tailored for each year’s cohort via a survey at the start of the fellowship year to identify interests and perceived needs of the cohort.	Each trainee has a mentoring team that includes a U.S. based mentor and at least one mentor from the host country. In addition, in 2020 the program included a junior mentor chosen from among the past 7 years of our Global Health Fellows and Scholars. Junior mentors provide opportunities for trainees to learn from the practical experiences of junior mentors acquired during their training period within the country, as well as provide an opportunity for the junior mentors to learn new mentoring skills under the guidance of more experienced mentors.	Trainee outcomes: 1. Upon completion of the training program, trainees will be familiar with modern principles of global health research 2. Trainees will develop and demonstrate skills necessary to pursue a career in global health research. Program objectives: 1. Provide a high quality research environment 2. Provide program support to trainees in program logistics 3. Provide support to nurture trainee interest in global health research. 4.Provide high quality interdisciplinary mentoring from both international and US-based mentors. 5. Provide professional development opportunities and support for mentors.	The program uses a variety of standardized program and mentor evaluations completed by trainees from each cohort: 1. Tracking and reviewing of multiple trainee performance indicators 2. Trainee pre- and post-fellowship self-assessment of competence in the global health research competencies 3. Mentor evaluation of trainee performance 4. Trainee evaluation of mentoring and program 5. Trainee and mentor evaluation of trainee’s impact on the research site.	Building an inclusive network across the nine collaborating countries that host trainees spanning 14 hours of time zones. Our solution was to develop a weekly series of calls covering core competencies and works-in-progress. Providing sufficient methodologic support and guidance on scientific writing to ensure each trainee is able to implement and publish the results of a mentored research project.	Weekly internet-based calls have provided opportunities to build a cohesive network of trainees who learn through peer review of each other’s projects. Calls have been adjusted each year to include more focus on scientific writing of manuscripts and grants and have incrementally increased the number of sessions that address development of leadership skills. Early in the fellowship period, many trainees had sufficient available time to participate in facilitated online courses in program management and leadership—skills we felt would be particularly useful for building careers in global health research.

**STAR Program Fellows** *https://www.ghstar.org/*	Mid-to-senior level global health professionals who play technical support roles at USAID or in secondment to Ministries of Health in LMICs 56 Fellows (20 US based, 36 overseas)* *Between program start and midway through Y2 (~18 months)	Not applicable	Hybrid design of facilitated peer-to-peer group mentorship with option to add 1-on-1 technical mentor pair. All STAR participants receive group mentorship for 6–10 sessions over 3–6 months. For Fellows paired with Technical Mentors, it is expected that the pair meets 3–4 times per year and their engagement is self-initiated.	The mentoring program will: 1. Reinforce all of the STAR core competencies and how they apply to individual work environments. 2. Promote partnering and collaboration [This links to larger program outcomes for STAR Learning and STAR overall] [10]	1. Annually, mentorship groups provide an opportunity for fellows to prepare for and/or discuss capacity building and knowledge sharing activities outside of STAR [assessed via regular check-ins]. 2. Mentoring groups will support fellows’ achievement of “practicing” level on core competencies by the end of the fellowship[assessed through endline competency assessment]. 3. Quarterly, fellows report that mentorship groups are good use of their STAR Learning time [assessed via regular check-ins].	Getting regular attention and attendance to mentorship groups. Establishing enough rapport to have thoughtful reflection. Ensuring that Technical Mentors actually meet mentees. Originally intended for mentorship groups to be facilitated by a GH expert, likely external to STAR, but it was difficult to balance the effort required by facilitation without actually incorporating the expert into the STAR team. Getting buy-in from on-site supervisor to take time away from work for mentorship group meetings.	Changed mentorship group meeting times from every 2 weeks to weekly to establish regularity of the group. Post-session monitoring of mentorship groups was too frequent and did not provide substantive feedback. Moved to evaluation survey after group meetings had ended. Making technical mentorship optional allowed for more opt-in approach, resulting in more commitment from fellows. Also reduced the burden on Technical Mentors who were experts external to the program. Using STAR facilitators for groups (rather than external experts) allowed for more control and consistency on content and delivery. Fellows have more time early on to dedicate to mentorship groups.


### ELM Program Participants and Partnerships

Despite different approaches, the programs we feature display several similarities. In all programs, the selected trainees were professionals with potential to develop as leaders in their fields over time. Additionally, the programs utilize an inter-professional training approach combining doctors, nurses, public health specialists, technical experts, interns, graduate students and academic faculty in various combinations. For example, EV4GH specifically targets young health policy and systems researchers, as well as other health systems actors such as activists, service managers, journalists and health practitioners, with the aim of producing a fundamental shift in addressing health systems. CHEPSAA, the only program featured here that was run from Africa, has a large scope of building multi-level capacity for African health policy and systems researchers and organizations. Their ELP is one component of these activities, and participants were chosen from within the CHEPSAA consortium.

Three out of the six programs featured collaborations and partnerships between trainees based in the US and those overseas, with the exception of Chatham House and CHEPSAA which were only open to Africans working in Africa, and EV4GH which is open to all applicants below 40 years of age with preference given to applicants from the Global South. At the time of writing this paper, the total number of trainees across these programs ranged from 14 for Chatham House to 290 for EV4GH, with most programs starting around 2010 and all programs still ongoing except CHEPSAA which closed in 2015.

#### Experiential learning

All programs, except STAR, incorporated experiential learning through placements in real life settings where the desired leadership skills could be gained and practiced, usually at a host organization in a LMIC. For example, Afya Bora ensures that participants are trained in a setting similar to where they will work, but different from their actual working stations to increase exposure to global health leaders and networks. While most programs prescribe the skills and competencies that one should acquire, the CHEPSAA program allows trainees to design their own program based on their perceived needs. Fogarty-NP conducts a survey at the beginning of the fellowship year that informs the curriculum each cohort will follow to gain the necessary skills and competencies, while STAR individualizes learning plans based on assessments. With respect to learning methods, we note a diverse array being employed, including online classes, didactic face-to-face sessions, workshops and meetings. For example, EV4GH uses a combination of online as well as in-person sessions that include case studies, simulations, plays and musical productions as a way to impart knowledge, build confidence, and provide opportunities to demonstrate skills.

#### Mentorship

Mentorship is provided throughout the course of the fellowships in all six programs through either peer-to-peer mentorship, pairing with a US-based mentor, pairing with a local mentor, or a combination of these. EV4GH leverages its global alumni network as mentors for the new cohorts. Mentoring is both face-to-face and remote, especially with mentors who are not local. For example, Chatham house program utilizes only virtual mentorship in its program. In some cases, such as with Afya Bora, trainees have a mentoring team at three different levels: a site mentor, a primary mentor and a country mentor. Mentors meet as often as necessary to fulfil the trainee’s learning needs, address challenges and monitor the progress on mutually agreed upon milestones in individual projects and competencies. Mentorship can be at the individual level or as a group—as in the STAR program where facilitated peer mentorship groups aim to support professional development and build connections between trainees. Fogarty-NP attempts to build capacity of new mentors, something that is critical in all settings, by pairing a junior mentor with a more experienced mentor.

### ELM Outcomes and Metrics

All of our featured global health leadership programs consider networking, interpersonal skills and collaborative relationships as part of their outcomes. Programs with a specific research component, such as Afya Bora, EV4GH, CHEPSAA, and Fogarty-NP, also explicitly link program outcomes to researcher skills and a nurturing research environment. Knowledge translation is mentioned explicitly for EV4GH and CHEPSAA, while STAR and Chatham House focus on leadership for global health programming and practice. Program outcomes were linked to and derived from each program’s objectives, and all programs include program-wide outcomes (e.g., enhanced networks and logistics support) as well as individual-level outcomes (e.g., gains in individual technical competencies and improved communication skills) (see ***Table 1***).

Self-assessments were a common component in all ELM programs’ approach to measuring outcomes. Several programs, namely STAR, Fogarty and Afya Bora, also instituted competency or skills assessments and checklists. Feedback from mentors and/or performance monitoring of site-based activities were included as part of Afya Bora, Chatham House, Fogarty-NP and STAR. CHEPSAA and EV4GH placed a strong emphasis on leadership-related activities measured through public presentations, event and team leadership, spearheading initiatives, and participation in research meetings; both programs also went through external evaluations. Although online learning is a component of all these programs, periodic meetings including all mentees and mentors have been used to address common challenges across the attachment sites, monitor progress, and foster face-to-face experience sharing. Overall, it was challenging to measure change in nebulous concepts related to trainees’ personal growth, such as leadership, especially for those that are more likely to manifest as trainees become alumni (e.g., becoming editor of a journal). Further research in this area is warranted.

### ELM Program Challenges and Lessons Learned

The six programs evolved independently and yet shared many of the same challenges during implementation and operation, with many leading to the development of similar solutions to these challenges. One common challenge was ensuring that program and mentoring expectations were clear and understood by both trainees and mentors. This challenge was often magnified through involvement of multiple institutions, countries and cultures, and flux of trainees and mentors across successive years. Most programs adopted a similar approach to ensuring program expectations were clear: holding discussions with trainees prior to or upon the start of each training year. Some also incorporated program expectations into learning contracts and individualized development plans (e.g., STAR). For programs using technical experts as mentors like Afya Bora, new technical experts often entered the program each year without prior knowledge of mentoring expectations, or with limited mentoring experience. Responses to this challenge varied, with some programs providing virtual sessions on mentoring to provide new mentors with expectations and mentoring tools to ensure a standardized approach that respected cultural and hierarchical differences across LMICs. Other programs assigned one experienced mentor affiliated with the program to each trainee or trainee group, who would also facilitate interaction with technical experts—thus ensuring these experts had a more defined role.

Another common challenge was establishing and maintaining rapport with trainees, who were often spread across the globe. Increased frequency of meetings was commonly adopted by many programs, including Fogarty-NP’s approach of weekly virtual meetings to promote trainee bonding. One logistical challenge related to virtual meetings and trainings was the instability of electricity and internet connections in some regions. An adaptation to this challenge was providing access to downloadable presentations and modules that could be viewed asynchronously, while simultaneously participating verbally via a low bandwidth application, such as WhatsApp platform.

A third common challenge was meeting the needs of trainees with different levels of technical capacity at the outset and with varied competency needs, while at the same time ensuring a diversity of trainee backgrounds, sustainability of training, and harmonization of the trainee’s research and practice activities with the needs and priorities of the country where they work. Most of these challenges were addressed through incorporating involvement of local mentors in the recruitment and selection of trainees, mentoring activities and management of the program.

Finally, these ELM programs represent substantial geographic diversity, and many are intentional about diversity in their recruitment and selection. However, regions such as Latin America and some Asian countries are under-represented in the programs featured here, despite their need for training support. For those programs that required travel across borders for training or project implementation (e.g., Afya Bora, EV4GH), delays or inability to obtain visas were also issues. This challenge has been amplified by the closure of country borders and embassies granting travel visas during the COVID-19 pandemic. All programs have pivoted to include more internet-based training—an adaptation that will likely continue in some form in future cohort years.

## Reflections from the Field

Here, we complement our personal experiences with ELM for global health leadership with experiences and recommendations from ELM program organizers and managers, mentors, funders and trainees, and also draw on data from monitoring and evaluation from featured programs^1^ to synthesize learnings thematically.

### Managing expectations

Explicit and clear goals and expectations of mentors as well as mentees were critical to managing expectations, minimizing misunderstandings (especially in culturally diverse programs), avoiding disappointment and enhancing potential for satisfactory program completion. As one mentee highlighted: *“The EV experience was not all ‘fairy tale’…It also required some critical ‘de-learning’ first, in order to ‘enable’ my mind for innovation and different ways of thinking and doing.”* (EV4GH Fellow). Another warned that,

*“if you get a mentor who did not get the orientation of Afya Bora…then, really, the fellow struggles a lot until they get what they need. Because, in our culture, the fellows cannot just go to their mentors and say, “you are not giving me what I am supposed to get,” so they politely accept that you are not getting you what you need.”* – Afya Bora

Unbalanced commitment leads to disappointment and often non-completion of the program target goals. ELM program managers, however, noted that managing expectations is not always easy given that different skills and baseline competence levels result in differences in speed of learning and growth, but may also produce frustration for trainees with lower skills at baseline. However, maintaining frequent contact with trainees can help overcome these frustrations and assist with managing expectations. STAR, for example, used a hybrid mentoring design that combined frequent meetings of peer mentor groups with less frequent meetings with an individual technical mentor in order to mitigate any unrealistic expectations of individuals’ mentor dyads.

### Diversity

The various programs demonstrate the value of diversity in the mentorship experience. This has led several of the ELM program organizers to stress that global health leadership programs need to be intentional in their recruitment and retention processes to ensure meaningful representation across demographics, such as gender, citizenship and discipline to attain the right mix for inter-professional learning. From the ELM organizer and trainee perspective, engagement across different institutions, disciplines and countries encourages a sense of comradery within the fellowship and provided opportunities for interdisciplinary learning. In reflecting on peer mentorship groups, a STAR fellow noted an *“Increased awareness of my learning needs and brought to light different contexts in which these competencies can be applied, as well as to accept that all of us are learning and that it never stops.”*

This diversity also leads to an expanded network of colleagues, mentors and friends, which was noted across respondents as invaluable. *“[My mentor] has helped me reexamine my assumptions…His support has very limited value add to my day-to-day work. However, his network and credentials as one of the experts in our space is something I plan to tap into.”* – STAR Fellow about their technical mentor

Funders also noted that global health leadership programs (from the North) usually target specific countries (e.g., fragile states, LMICs), which limits participation, reduces diversity and neglects the presence of many versions of the Global “South.” It is unclear where to fit countries such as former Soviet Republics or Latin American countries which are ranked middle or high-income by the World Bank for programs such as these.

### Appropriate content and approaches

Global health programs need continuous reflection about not becoming a new way of colonization from the North to the South. Specifically, as ELM components present leadership skills as envisioned or experienced in the “Global North,” these may not fit in other cultures or other settings. For example, over-emphasis of learning on presentation skills at the cost of listening and participation skills. For trainees, skill building in emerging areas (e.g., blogging or social media) may not align with historical academic incentive structures that emphasize academic writing above all else. As noted by one mentee *“[Project final reports] are a great part of the fellowship; however, the direction seemed so scientific in nature with a drive to publish…If one did an implementation project at an attachment site, I’m not sure they would write a report successfully in that format.”* – Afya Bora Fellow

Attention to cultural variations across countries regarding the approach to mentorship and training increases the likelihood of effective cross-institutional collaboration and implementation of new programs within existing hierarchies. Several programs featured here, such as Afya Bora, CHEPSAA and Fogarty-NP, allow for customizing of trainee learning; still, program content need to be tailored to the context, reflecting the local values and different ways of life.

### Sustainability and the social return on investment

Although altruism and a desire to grow the field of global health professionals may be insufficient to garner the necessary commitment, all parties in a mentoring relationship have to find a value-add and meaningful incentives for continued engagement as captured here: *“I think another thing that has changed over time is…when I realized it is important to have fellows be involved in the recruitment process, so they get HR experience. Because that is something you don’t learn in school, how to do HR” –* Afya Bora mentor. Similarly for trainees, the continued success of the program can include incentives similar to that of EV4GH and Fogarty-NP, which encourage alumni to become mentors for the future cohorts. Incorporating program alumni into the integration and mentoring of new trainees is highly desired by both incoming and graduating trainees and provides practical opportunities for alumni to practice leadership skills acquired through the training program. This is appealing to funders as well as leading global health organizations given that as cohorts graduate and become global health leaders, they take on roles in funding and partner institutions where their training can influence the direction institutions take: *“[EV4GH] ‘Forces’ the community to think in generational terms, that also causes them to take capacity building more seriously.”* – EV4GH Organizer

### Reflections on Program Design

Our review of ELM activities within global health leadership programs raised critical considerations that should be reflected on and addressed during program design. While they warrant more exploration than we can offer here, we touch on them briefly to emphasize their importance and their inter-related nature for all ELM programs. These topics are also covered in more depth elsewhere in this Collection.

***Ethics:*** Brain drain, reflecting trainees’ within-country or international migration to higher-resourced areas, is a persistent challenge. Programs should actively avoid brain drain while ensuring that the program’s needs do not disrupt activities of local organizations; this often benefits from explicit communication between the North-South leadership regarding alumni support. Also, environmentally speaking, short in-person meetings of participants not located in close proximity may no longer be ethical; increased availability of internet-based communication programs have made virtual face-to-face mentoring possible in most settings.

***Technology:*** Internet-based tools allow people to communicate across greater distances, but with a loss to the personal connection and relationships that develop through face-to-face meetings. One mitigation strategy is increasing support and interpersonal interactions in the local environment. As a result of the COVID-19 pandemic, inclusion of virtual platforms and approaches to training has produced new opportunities for innovation and cost savings, and inclusion of more geographically diverse trainees and mentors.

***Gender:*** Global health leadership programs have created new opportunities for people and specialties with persistent gender biases that have not traditionally been targeted for training, such as nurses, midwives, architects, lawyers and social workers. Intentional consideration of gender is required throughout all stages of the program—from recruitment to trainee and mentor selection and alumni support—to ensure inclusion of diverse voices, increase innovation and generalizability.

***Age:*** Many programs aim to train the next generation of global health leaders with preference for junior or young trainees. Although some consider this biased, professional opportunities traditionally recruit younger trainees as they have a longer anticipated period of working as future leaders. However, career paths develop at different speeds around the world, creating an imbalance, especially for researchers in the Global South, where despite achieving high levels of training, leadership opportunities may be limited to more senior colleagues. Also, Engaging more senior trainees who are already employed within the health system may provide new opportunities to implement change within existing systems to enhance research, leadership and training.

***Framing:*** Historically, most global health leadership programs have been designed, developed, managed and implemented with significant input and/or funding from the Global North. As such, these programs face pitfalls related to the complex dynamics whereby “high capacity” countries (typically Northern ones) became the supplier of training to “lower capacity” Southern ones. Even programs with administrative bases partly in the South, such as Afya Bora and EV4GH, may not have sufficient LMIC perspectives in their framing of successful leadership training or outcomes of such training. CHEPSAA may be a positive anomaly in this arena, in that it permitted aspiring leaders to design their own program of capacity building based on their own perceived competency needs. Critical conversations are needed about how to better integrate LMIC contexts, ideations and practices of what leadership means at the conception stage; how evolving forms of leadership (e.g., transformative approaches) can engage with the realities of hierarchical/directive systems; and how emerging leadership programs can be framed within the landscape of North-to-South leadership transitions.

## Conclusion

The COVID-19 pandemic has challenged the structure and ideas of global health governance and drawn attention to the role of global health leaders. Now more than ever, people inside and outside global health recognize the power struggles, alliances and abilities required to reach public health goals. They also recognize that health is a personal, social and planetary good that requires the coordinated action of many actors, guided, pushed or accompanied by competent leaders. Thoughtful design and delivery of ELM programs to ensure the next generation of global health leaders can successfully navigate national and international challenges with sensitivity and awareness, while maintaining a broad diversity of cultures, disciplines and training needs should ensure that these future leaders are able to examine and take appropriate and effective action when confronting the big and small challenges they will encounter on a daily basis.

We believe that integration of ELM into global health leadership programs, despite the challenges noted, provides important and unique tools to respond to the current global crisis – especially through building and strengthening networks of global health professionals and scientists. Given this value, it is imperative to sustain existing ELM programs and further strengthen these and emerging programs to ensure they are adapted to address local challenges and health conditions, emphasize inclusion of locally selected trainees who can later serve as junior mentors, and maintain international collaboration through sustained mentorship relationships. Such efforts can strengthen the collaboration and joint efforts needed to address current and future global threats.

## Footnotes

Since these reflections are drawn from existing programmatic monitoring efforts, ethics review was not applicable.
